# PCR-based Sepsis@Quick test is superior in comparison with blood culture for identification of sepsis-causative pathogens

**DOI:** 10.1038/s41598-019-50150-y

**Published:** 2019-09-20

**Authors:** Ngo Tat Trung, Nguyen Sy Thau, Mai Hong Bang, Le Huu Song

**Affiliations:** 1 Vietnamese-German Center for Medical Research (VG-CARE), 108 Military Central Hospital, Hà Nội, Vietnam; 2 Centre for Genetic Consultation and Cancer Screening, 108 Military Central Hospital, Hà Nội, Vietnam; 3 Faculty of Tropical and Infectious Diseases, 108 Military Central Hospital, Hà Nội, Vietnam; 4 Department of Molecular Biology, 108 Military Central Hospital, Hà Nội, Vietnam

**Keywords:** Biological techniques, PCR-based techniques

## Abstract

Sepsis is an acute, often fatal syndrome that requires early diagnosis and proper treatment. Blood culture (BC) is the gold standard for the identification of pathogens, however it has marked limitations, including that it is time-consuming (delaying treatment) and can only detect microbes that readily grow under culture conditions. Alternatively, non-culture-based methodologies like polymerase chain reaction (PCR) are faster but also have limitations; e.g., the reaction is often inhibited by the abundance of human DNA and thus can only detect limited known target pathogens. In our previous publication, we have demonstrated a proof-of-concept of a simple pre-analytical tool to remove human DNA from patients’ blood specimens, hence allowing downstream PCRs to detect rare bacterial genetic materials. In the current study, we reported a better performance of a novel prototype diagnosis kit named Sepsis@Quick that combines human DNA removal step with real-time PCRs compared to blood-culture for identifying sepsis causative bacteria. Our data showed that Sepsis@Quick is superior to blood culture in which the novel diagnostic kit could identify more pathogens and even polymicrobial infection, faster and less influenced by the empirical administration of broad spectrum antibiotic therapy (single administration or combination of cephalosporin III and fluoroquinolon). Additionally, for the first time, we demonstrated that positive results achieved by Sepsis@Quick are significantly associated with a reduction of sepsis-related mortality.

## Introduction

Sepsis is among the most common causes of mortality for hospitalized patients worldwide, and its incidence is steadily increasing^1,2^. So far, blood-culture is commonly known as the gold standard for the detection of microbial pathogens in the bloodstream. This method, however, possesses some intrinsic limitations, as it is laborious and can only identify microbes that grow under optimized culture conditions^3,4^. Volumes of 20–30 ml of blood are required for aerobic and anaerobic microbial cultures^5,6^, such volumes may be a challenge to obtain from elderly or neonatal patients. The classical method is also difficult in establishing the diagnosis for patients who uptake antibiotics before^7^. In clinical practice, only 14–30% of sepsis cases are clinical-relevantly diagnosed by blood culture^8,9^. Therefore, it is essential to establish a rapid diagnostic procedure to complement, or even improve the performance of blood culture approaches.

**Figure 1:**
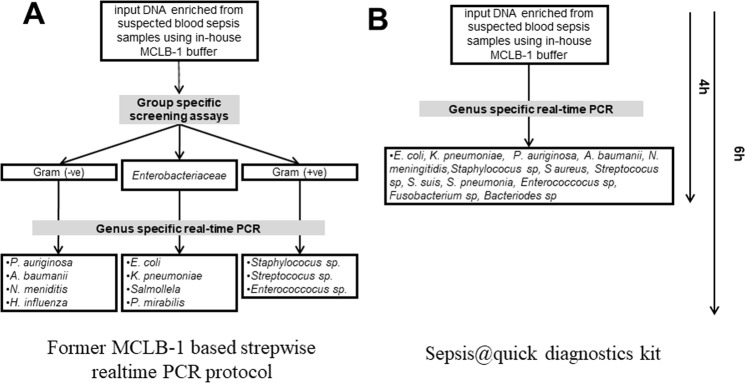
Scheme of study design and workflow. (**A**) Former MCLB-1 based stepwise realtime PCR protocol including Group-specific screening by PCR reactions targeting bacterial 16SrRNA gene to differentiate Gram-positive, Gram-negative and *Enterobacteriaceae* groups. Samples positive in the screening assay were subjected to genus-specific real-time PCR reactions to detect 13 most common sepsis causative pathogens. (**B**) the current protocol of Sepsis@quick diagnostics kit was subjected to individual to genus-specific real-time PCR of *E*. *coli*, *K*. *pneumoniae*, *P*. *auriginosa*, *A*. *baumannii*, *N*. *meningitidis*, *Staphylococus sp*, *S aureus*, *Streptococus sp*. *S*. *suis*, *S*. *pneumonia*, *Enterococcocus sp*, *Fusobacterium sp and Bacteriodes sp*.

Polymerase Chain Reaction (PCR) based techniques can sense small amounts of pathogen’s DNA directly from blood samples within 3–6 hours, thereby supporting a treatment afterward^10^. However, PCR-based diagnosis may significantly be thwarted by human DNA abundance and DNA fragments which are evolutionary conserved between humans and bacteria^11–15^. Recently, we demonstrated as a proof of concept that the depletion of human DNA from blood samples of septic patients prior to the PCR based diagnostic steps enhances the detection threshold up to 10 CFUs of *E*. *coli* per ml. By this assay, we showed an enhanced detection of up to 40% compared to 23% of blood cultures^16^. It was a promising result providing a new tool for septic diagnosis of Infectious disease specialists. However, in clinical practice, we realized that the group-specific primers/probes designed for Gram-negative, Gram-positive and *Enterobacteriacea* bacteria acquired unsatisfied performance. As a consequence, we omitted the group-specific screening and subjected the DNA-depleted specimens directly as samples for individual genus-specific real-time PCR assays which target common sepsis-causative pathogens, namely *Escherichia coli*, *Klebsiella pneumoniae*, *Pseudomonas aeruginos*, *Acinetobacter baumanii*, *Neisseria meningitidis*, *Staphylococus sp*, *Staphylococus aureus*, *Streptococus* spp., *Streptococcus suis*, *S*. *pneumonia*, *Enterococcocus* spp., *Fusobacterium* spp. *and Bacteriodes* spp. (Fig. 1). This modification now translated into a prototype sepsis diagnostic kit (so-called Sepsis@Quick) and is intellectually protected under Vietnamese national filed patent number 17556. In addition, during our clinical practice, we also documented some patients who were treated with antibiotics prior to blood-sampling for laboratory diagnosis, often got negative blood-culture results while their corresponding Sepsis@Quick based diagnostics were positive. Therefore, it prompted us to conduct this prospective observational study to comparatively evaluate the clinical uses of our novel Sepsis@Quick kit and conventional blood cultures for the diagnosis of bloodstream infection.

## Results

### Characteristics of the study cohort

The characteristics of the study population according to the Sepsis-3 classification were shown in Table 1. In total, 144 patients were included: central nervous system infection (CNS, 24), pneumonia (34), intra-abdominal infection (39), urinary tract infection (18), skin-soft tissue infection (16) and unknown source (13). The average SOFA score was 7(4–10) (median (IQ)) and average APACHE II score was 17.2 ± 7.2. Among 144 study subjects, 125 patients had serological lactate measurements with average concentrations (median (IQ)) of 3.4 (2.1–6.2) mmol/L. 43 patients received continuous renal replacement therapies (CRRT); 81 patients were treated with antibiotics before blood-sampling for detecting pathogens and 74 out of those patients acquired antibiotic revision latter. Most of them were treated by antibiotics longer than 1 week before hospitalized (1 to 10 days) and cephalosporin III were more frequent used. 62 patients were diagnosed as septic shock and 64 patients had other comorbidities, including cancer, diabetes mellitus, HIV/AIDS, hypertension, congestive heart failure, coronary disease, chronic renal failure, chronic hepatic failure, chronic alcohol abuse and chronic obstructive pulmonary disease. The most frequent pathogens detected were *Escherichia coli*, *Klebsiella pneumoniae*, *Streptoccocus* spp. and *Neisseria meningitidis*; the time-frame for blood culture reading was 48 hours, whereas it took 4 hours only for Sepsis@Quick to establish the diagnosis.

**Table 1:** Clinical and laboratory characteristics of the study cohort.

Characteristics	All patients (n = 144)	Blood culture			Sepsis@quick		
		*Pos* (*n* = *49*)	*Neg* (*n* = *95*)	*p*	*Pos* (*n* = *83*)	*Neg* (*n* = *61*)	*p*
Age (year)	61.4 ± 17.4	62.4 ± 14.5	60.8 ± 18.5	0.*88*	60.3 ± 17.6	63.1 ± 17	*0*.*43*
Male, number (%)	106 (73.6)	32 (65.3)	74 (77.9)	*0*.*10*	59 (71.1)	47 (77.1)	*0*.*42*
**Source of infection**, **number (%)**							
CNS	24 (16.7)	7 (14.3)	17 (17.9)	*0*.*58*	17 (20.5)	7 (11.5)	*0*.*15*
Pneumoniae	34 (23.6)	7 (14.3)	27 (28.4)	*0*.*06*	13 (15.7)	21 (34.4)	*0*.*009*
Intra-abdominal	39 (27.1)	16 (32.7)	23 (24.2)	*0*.*28*	26 (31.3)	13 (21.3)	*0*.*18*
Urinary tract	18 (12.5)	11 (22.4)	7 (7.4)	*0*.*01*	12 (14.5)	6 (9.8)	*0*.*41*
Skin-soft tissue	16 (11.1)	4 (8.2)	12 (12.6)	*0*.*42*	9 (10.8)	7 (11.5)	*0*.*91*
Unknown	13 (9.0)	4 (8.2)	9 (9.5)	*0*.8^*^	6 (7.2)	7 (11.5)	*0*.38^*^
Other comorbidities, Number (%)	64(44.4)	18 (36.7)	46 (48.4)	*0*.*18*	33 (39.8)	31 (50.8)	*0*.*19*
Shock, number (%)	62 (43.1)	25 (51.0)	37 (38.9)	*0*.*17*	35 (42.2)	27 (44.3)	*0*.*8*
Immunosuppression, number (%)	29 (20.1)	8 (16.3)	21 (22.1)	*0*.*41*	12 (14.5)	17 (27.9)	*0*.*047*
Antibiotic before blood culture, number (%)	81 (56.3)	22 (44.9)	59 (62.1)	*0*.*049*	47 (56.6)	34 (55.7)	*0*.*92*
Number of dysfunction organ, mean ± SD	3.2 ± 1.2	3.2 ± 1.3	3.1 ± 1.15	*0*.*26*	3.2 ± 1.3	3.4 ± 1.4	*0*.*74*
APACHE II, mean ± SD	17.2 ± 7.2	17.4 ± 7.9	17.5 ± 7.9	*0*.*97*	16.47 ± 8.1	18.4 ± 7.1	*0*.*087*
SOFA, median (IQ)	7 (4–10)	8 (5–10)	7 (4–10)	*0*.*32*	7 (4–10)	8 (4–10)	*0*.*88*
Mechanical ventilation, number (%)	61 (42.4)	23 (46.9)	38 (40.0)	*0*.*43*	29 (34.9)	32 (52.5)	*0*.*036*
CRRT, number (%)	43 (29.9)	13 (26.5)	30 (31.6)	*0*.*53*	20 (24.1)	23 (37.7)	*0*.*08*
PCT, median (IQ), (ng/ml)	37.7 (8.7–100)	67 (15.8–100)	35.6 (7.3–96.1)	*0*.*033*	61.8 (20.6–100)	13.9(5–56.9)	*0*
Leukocyte count, mean ± SD, (G/l)	16.6 ± 10.4	16.5 ± 10.6	16.6 ± 10.4	*0*.*95*	17.1 ± 10.1	15.9 ± 10.9	*0*.*25*
Lactate, median (IQ), (mmol/l)	3.4 (2.1–6.2)	4.5 (3–10.3)	2.7 (1.9–4.9)	*0*.*001*	3.5 (2.4–6.3)	2.8 (1.7–6.0)	*0*.*18*

Abbreviation: CNS (Centrel nervous system), APACHE II (Acute Physiology and Chronic Health Evaluation II), SOFA (Sequential Organ Failure Assessment), CRRT (Continous Renal Replacement Therapy), PCT (Procalcitonin), IQ (Interquartile). Data were presented as mean ± SD, median with interquartile range or percentage where appropriate.*: Fisher’s exact test.

### Capacity of pathogenic detection of Sepsis@Quick and blood culture

**Figure 2:**
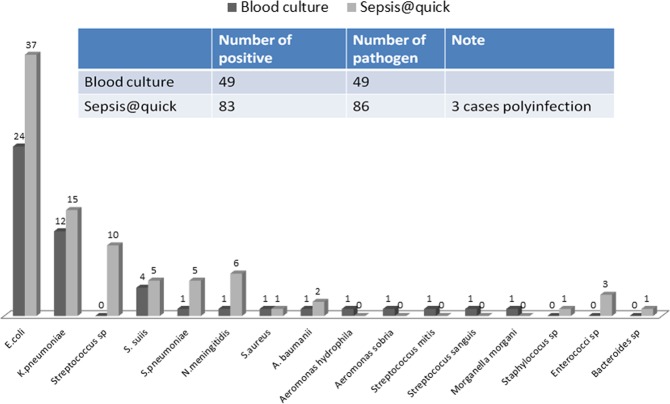
Numbers of individual pathogens detected by blood culture and Sepsis@quick: Upper panel shows the numbers positive cases or number of polymicrobial infection detected by blood culture or sepsis@quick, lower panel shows number of individual microbial pathogens detected by blood culture or sepsis@quick.

**Figure 3:**
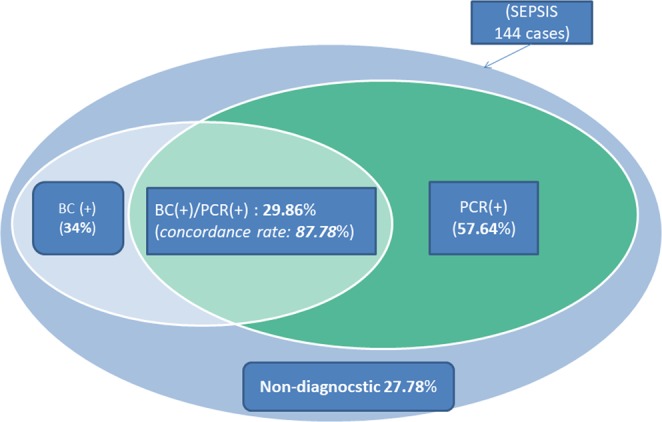
Diagnostic performance and concordance portion between the two methods: Total 144 patient blood samples were recruited into this current study, of which, Sepsis@Quick identified 83/144 (57.64%) positive. Blood culture approach detected 49 cases (34%) positive and 43 out of 49 culture-positive cases (87.8%) were co-detected by Sepsis@Quick and only 40 cases (27.78) were not diagnosed.

By using Sepsis@Quick test, 83 out of 144 recruited samples (57.6%) were identified pathogens including (*E*. *coli* (37), *K*. *pneumoniae* (15), *N*. *meningitidis* (6), *S*. *suis* (5); *S*. *pneumoniae* (5), *S*. *aureus* (1), Staphyloccocus sp (1), *Streptoccocus* spp. (10), *Bacteriodes* spp (1), *Enterococcocus* spp (3), *A*. *baumannii* (*2*). In addition, three out of 83 (3.6%) samples were detected with polymicrobial infection by Sepsis@Quick (Fig. 2). The conventional blood culture approach achieved positive colonies from 49 cases (34%); consisting of *E*. *coli* (24), *K*. *pneumonia* (12), *N*. *menintigitis* (1), *S*. *suis* (4); *S*. *pneumonia* (1), *S*. *aureus* (1), *S*. *mitis* (1), *S*. *sangius* (1), *A*. *hydrophilia* (1), *A*. *sobria* (1), *M*. *morgani* (1) and *A*. *baumannii* (*1*), (Fig. 2); 43 out of 49 culture-positive cases (87.8%) (Fig. 3) were also detected by Sepsis@Quick. *E*. *coli* and *K*. *pneumonia* were the most frequently detected pathogens by both approaches. Detailed patients’ characteristics according to Sepsis@Quick and blood culture results are shown in Table 1.

### Influence of prior antibiotic treatment on diagnostic performance of blood culture and Sepsis@Quick

Our data revealed that prior initiation of antibiotic treatment significantly reduced positivity rates acquired by blood cultures (from 42.9% to 27.2%; P = 0.049), while diagnostics results achieved by Sepsis@Quick did not differ between naïve patients (57.1%) and antibiotics-treated patients (69.1%) (P = 0.92), (Table 2).

**Table 2:** Influence of previous treatment with antibiotics on diagnostics performance of Sepsis@quick and blood culture.

Methods		Previous antibiotic administration		P	OR (95%CI)
		Yes, n (%)	No, n (%)		
Blood culture	Pos	**22 (27**.**16%)**	**27 (42**.**86%)**	***0***.***049***	**0**.**497 (0**.**247–1**.**0)**
	Neg	59 (72.84%)	36 (57.14%)		
Sepsis@quick	Pos	47 (69.14%)	36 (57.14%)	*0*.*92*	1.04 (0.53–2.02)
	Neg	34 (30.86%)	27 (42.86%)		

### Sepsis@quick influences better to the revision of antibiotics therapies

Admitted patients who were diagnosed with either severe sepsis or septic shock would be treated with empirical broad-spectrum antibiotics such as cephalosporin III or/and fluoroquinolon or/and aminoglycoside, carbapenem → cephalosporin III or carbapenem or/and fluoroquinolon or/and aminoglycoside) before or without the identification of causative microbial pathogens. And once, causative pathogens were identified, the previously indicated antibiotics would be revised. In our study cohort, we documented that the revisions of antibiotic therapies happened to 74 cases. As seen in Table 3, sepsis@Quick provided positive diagnostics for 45 cases, whereas blood culture only detected microbial pathogens only in 26 scenarios (P = 0.0017); Especially, amongst 20 cases for whom, the antibiotic de-escalation were indicated, Sepsis@Quick identified positive results for 15 cases, which is significant higher that detected by blood culture (8 cases, P = 0.025).

**Table 3:** Influence of Sepsis@quick on revision of antibiotic administration.

Methods	Changes in antibiotic therapies			De-escalation of antibiotic therapies		
	Positive n (%)	Negative n (%)	P	Positive n (%)	Negative n (%)	P
Sepsis@quick	45 (60.8%)	29 (39.2%)	**0**.**0017**	15 (75%)	5 (25%)	**0**.**025**
Blood culture	26 (35.1%)	48 (64.9%)		8 (40%)	12 (60%)	

### Sepsis@Quick diagnosis performance reduces sepsis-related mortality

**Figure 4:**
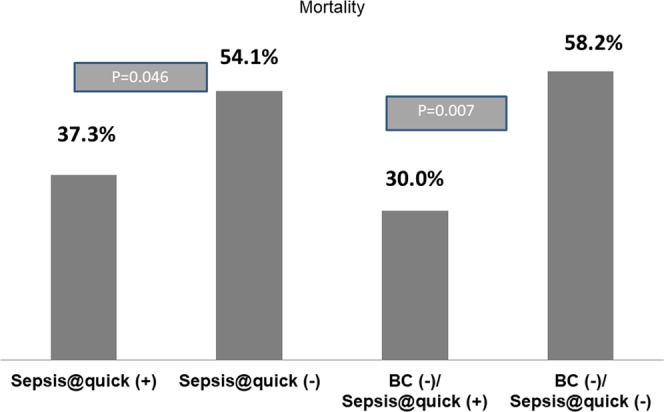
Relationship between sepsis-related mortality rate and diagnostic performance of Sepsis@quick: Two left bars show comparisons of mortality rate between patient group carrying sespsis@quick positive result versus patient group with sespsis@quick negative diagnosis. Two right bars show comparisons of mortality rate between patient group of blood culture negative but sepsis@quick positive versus patient group with blood culture negative and sepsis@quick negative (double nengative). Both comparison were performed by Chi-square test.

Our data reveals that even though the SOFA score^17^ (most comprehensive criteria for organ failure evaluation of sepsis patients) was not significantly different between the two subgroups (Sepsis@quick (+), n = 83 versus (Sepsis@quick (−), n = 61) or between two subgroups (BC(−)/Sepsis@quick (+) n = 40) versus (BC(−)/Sepsis@quick (−) n = 55), (Supplementary Fig. 1) the mortality rates were clearly distinguishable between of Sepsis@quick (+) (n = 83, 37.3%) versus Sepsis@quick (−) (n = 61, 54.1%), p = 0.046 and especially in patient group with blood culture negative, the use of sepsis@quick really associated with the reduction of sepsis related mortality rate (BC(−)/Sepsis@quick (+) n = 40, 30%) versus (BC(−)/Sepsis@quick(−) n = 55, 58.2%) p = 0.007 (Fig. 4).

## Discussion

Currently, blood culture and DNA based PCR methods are the main tools for identification of sepsis causative pathogens. However, while blood culture is time-consuming and has other drawbacks, including the difficulty to apply optimal growth conditions for unknown or/and fastidious pathogens, large volumes of blood required for aerobic and anaerobic cultures^5,6^, impairment of cultures due to pre-treatment with antibiotics, the direct identification of pathogens by PCR is difficult due to low concentrations of bacteria in blood samples and the strongly inhibitory effect of human DNA. Hence, in daily clinical practice, diagnostics results gained by the two methodologies are not frequently consistent.

Our research group and Oliver Liesenfeld *et al*. have already demonstrated a proof-of-concept that by reducing the amount of human DNA from septic patients’ blood helps to significantly enhance the detection limit of the downstream PCRs in the diagnosis of blood stream infection^15,16^. In this study, we modified a previous assay to formulate a prototype diagnosis kit called Sepsis@Quick and evaluate the clinical performance of this novel kit against that of conventional blood culture for identification of sepsis causative pathogens.

Our data showed that Sepsis@Quick testing was superior to blood cultures. With the use of Sepsis@Quick, more pathogens and even polymicrobial infection were identified. The Sepsis@Quick’s performance was faster and less influenced by previous antibiotic treatment. Moreover, positive results achieved by Sepsis@Quick in a shorter diagnostic time provided additional awareness that helps clinicians to revise the previous use of antibiotics or/and drive the changes in therapeutics practices properly; thereby indirectly reducing the sepsis-related mortality rate. In the present study, blood cultures were able to establish a diagnosis for 34% of recruited patients; this is in the range gained by other studies^18,19^. In contrast, Sepsis@Quick could provide diagnoses for 57.6% of the study group. In addition, more than 87% of blood-culture positive cases were confirmed by the Sepsis@Quick system. In other studies, results obtained by another PCR-based system (SeptiFast) were quite variable while the SeptiFast assay had a higher diagnostic performance compared to blood culture^20–24^. Whereas, both methods displayed either similar sensitivities or the SeptiFast assay even acquired less favorability with respect to blood culture^13,19,25,26^.

Despite the recommendations from the Surviving Sepsis Campaign on archiving blood samples for culture prior to treatment with broad-spectrum antibiotics^27^, about 50–70% of septic patients receive antibiotics before blood samples are collected for culture^28^. Under antibiotic pressure, it is hard for some bacteria to form colonies, hence reducing the effectiveness of blood cultures, considering this and keeping in mind that blood-culture normally provides a diagnosis after 48 hrs or even longer, there would be no result available after four hours upon patient recruitment if only the blood-culture approach is used in clinical practices. In our study, the identification of sepsis-causing pathogens by Sepsis@Quick kit was finalized as quickly as four hours but our molecular approach is an open system that allows the users to economically customize it with a number of pathogens wanted relevant to given local medical settings. Users of the Sepsis@Quick system do not need to process patient samples in batches mode but can conduct the assay for individual patients upon the collection of samples, ensuring the availability of Sepsis@Quick results in four hours; to this point, other similar studies also support our findings. In the previous studies, the SeptiFast kit was used to detect the pathogens significantly earlier than blood cultures^19,20^. However, we are the first to demonstrate that positive results provided by PCR-based approaches help to decrease sepsis-associated mortality. In another aspect, although, Sepsis@Quick identify a narrow range of detectability than SeptiFast does, SeptiFast is a closed system with a given number of detectable pathogens^13^, whereas Sepsis@Quick is an open system, allowing ones to customize for the detection of pathogens relevant for given medical settings^29^.

Nevertheless, there are several limitations in the present study: it was a single center observational study including a high proportion of sepsis shock patients (n = 62.4%) who might carry higher densities of bacterial loads in the peripheral blood; this might limit the generalizability of the findings. However, the results are consistent with those generated by other investigators, who also compared the performance of PCR versus blood culture for identification of sepsis causative pathogens^20,22–24^, suggesting that these findings may apply to other patient cohorts as well. Our current version of Sepsis@Quick assay cannot detect fungi and other bacterial pathogens such as *Aspergillus fumigatus*, *Candida* spp., *Burkholderia* spp., *A*. *hydrophilia*, *A*. *sobria*, *M*. *morgani*, *E*. *aerogenes/cloacae*, *M*. *morganii* and *S*. *marcescens*. This may partially explain the lack of DNA amplification in about 14% of episodes, whereas blood culture was still able to show growth. Hence, inclusion of more pathogens into the future forms of Sepsis@Quick is needed to improve the pathogenic detection spectrum.

## Conclusions

The novel PCR-based Sepsis@Quick system provides faster detection and identifies more bacterial pathogens than the conventional blood culture, particularly in patients who undergo previous antibiotics treatment, hence reducing sepsis-related mortality.

## Methods

### Study cohort and patient blood sampling

A total of 144 blood samples from naïve and septic patients pre-treated with antibiotics were collected at the 108 Military Central Hospital, Vietnam. Diagnoses of sepsis were confirmed by physicians in accordance with an acute increase in Sequential Organ Failure Assessment (SOFA) scores by two or more as defined by the Sepsis-3 guidelines^17^. Further characteristics of the patients (age, sex, comorbidity, immunosuppression, the SOFA scores and signs of organ dysfunction were documented at admission (Table 1)^17^.

### Study design

All methods used in this study were in accordance with the relevant guidelines and regulations and were approved by the institutional review board and an independent Ethics Committee of the 108 Military Central Hospital, Hanoi, Vietnam. Informed written consent was obtained from all study patients. Twelve milliliters of venous blood collected in duplicates from all patients were subjected to both blood culture and the Sepsis@Quick test in order to identify the causing pathogen(s) (Fig. 1).

### Preparation of reagents under aseptic conditions

For decontamination of the PCR master mix, 8-methoxypsoralen was dissolved in dimethylsulfoxide (DMSO) (Sigma-Aldrich, St. Louis, MO, USA). For decontamination of the real-time PCR master mix reagents, 25 µg/mL of 8-methoxypsoralen (Sigma-Aldrich) and 10 min of exposure to UV irradiation at 366 nm over a distance of three cm were applied^30^. Components included in the Sepsis@Quick kit were also decontaminated by UV irradiation at 280 nm over a distance shorter than five cm^30^. Working places, as well as all accessories and tools, routinely undergo weekly decontamination by spraying with DNA-ExitusPlus™ solution (AppliChem, Darmstadt, Germany).

### Enrichment of bacterial DNA

Enrichment of bacterial DNA for downstream PCR analysis was performed similarly to our previous study with minor modifications^16^. In brief, 2 volumes of 1.2 ml peripheral venous blood samples obtained from every sepsis patients recruited were added to an equal volume of mammalian cell lysis buffer (MCLB-1, containing Na2CO3 and Triton-X100) for three minutes at 37 °C to allow for shearing of human chromatin into DNA fragments. After the incubation step, an equal volume of neutralization buffer (NB buffer containing Tris–HCl) was added in order to prevent further cell lysis. The samples were then centrifuged at 5000 g for 5 min. Supernatants were discarded and bacterial pellets were reconstituted in 200 μl of 1x Tris buffered saline (TBS, pH 7.6) and used for isolation of bacterial DNA by the Automatic Nucleic Acids Extraction System (Sacace Biotechnologies, Como Italy). Eluted DNA was reconstituted in 200 ul 25 mM Tris–HCl pH8.0 for downstream PCR analysis (MCLB-1 and NB buffer are included in the Sepsis@Quick kit).

### Real-time PCR conditions

The real-time PCR assay mixtures consisted of 7.5 µl Taqman real-time PCR master mix (Qiagen, Hilden, Germany), 5 µl of DNA template, 5 pmol of primers and 0.2 pmol of probes (included in the Sepsis@Quick kit). Reactions were run in the Stratagene M3000p (San Diego, CA, USA) device with a pre-incubation step at 50 °C for 15 min, initial denaturation at 95 °C for 5 min, followed by 45 cycles of 95 °C for 15 sec and 60 °C for 60 sec.

### Automatic bacterial culture

Two volumes of collected blood samples from each patient were subjected for blood culture in the BD BACTEC^TM^ 9120 system (Becton Dickinson, New Jersey, USA) at 36 °C ± 0.5 °C for 48 hours. Once the growth occurred in both tubes, identification of bacterial species was performed using the VITEK® 2 automated system (BioMérieux, Marcy-l’Étoile, France).

### Statistical analysis

All statistical analyses were performed using the SPSS software (v19). Values are given as numbers with percentages and median with interquartile where appropriate. Chi-square or Fisher’s exact tests were performed to compare categorical variables and positivity rates of the different diagnostic test systems. Mann-Whitney U or Kruskal–Wallis was used to compare two or more means, respectively. Sensitivity and specificity of the Sepsis@Quick system were calculated by using the blood culture positive result or Sepsis-3 criteria as reference^17^. The odds ratio and 95% confidence intervals (CIs) were calculated in order to estimate the association of negative results of both systems with previous exposure to antibiotics; The level of statistical significance was set at 0.05 and all tests were 2-tailed.

## Supplementary information

Supplementary information

## Data Availability

Data and supporting materials associated with this study will be shared upon request.

## References

[1] Knoop ST, Skrede S, Langeland N, Flaatten HK (2017). Epidemiology and impact on all-cause mortality of sepsis in Norwegian hospitals: A national retrospective study. PLoS One..

[2] Herran-Monge, R. *et al*. Epidemiology and Changes in Mortality of Sepsis After the Implementation of Surviving Sepsis Campaign Guidelines. *J Intensive Care Med*. 885066617711882 (2017). 10.1177/0885066617711882 28651474

[3] Gerdes JS (1991). Clinicopathologic approach to the diagnosis of neonatal sepsis. Clin Perinatol..

[4] Gerdes JS (1994). Clinicopathologic approach to the diagnosis of neonatal sepsis. Isr J Med Sci..

[5] Nicasio Mancini (2010). The era of molecular and other non-culture-based methods in diagnosis of sepsis. Clin Microbiol Rev..

[6] Wilson, M. L. Principles and procedures for blood cultures. Approved guideline, Clinical and Laboratory Standards Institute. *CLSI*. (2007).

[7] Scheer CS (2019). Impact of antibiotic administration on blood culture positivity at the beginning of sepsis: a prospective clinical cohort study. Clin Microbiol Infect..

[8] Frank Bloos (2012). Evaluation of a polymerase chain reaction assay for pathogen detection in septic patients under routine condition: an observational study. PLoS One..

[9] Jordan JA, Durso MB, Butchko AR, Jones JG, Brozanski BS (2006). Evaluating the near-term infant for early onset sepsis: progress and challenges to consider with 16S rDNA polymerase chain reaction testing. J Mol Diagn..

[10] Ishmael FT, Stellato C (2008). Principles and applications of polymerase chain reaction: basic science for the practicing physician. Ann Allergy Asthma Immunol..

[11] Wendy LJ (2009). Evaluation of new preanalysis sample treatment tools and DNA isolation protocols to improve bacterial pathogen detection in whole blood. J Clin Microbiol..

[12] Gebert S, Siegel D, Wellinghausen N (2008). Rapid detection of pathogens in blood culture bottles by real-time PCR in conjunction with the pre-analytic tool MolYsis. J Infect..

[13] Von Lilienfeld-Toal M, Lehmann LE, Raadts AD, Hahn-Ast C, Orlopp KS (2009). Utility of a commercially available multiplex real-time PCR assay to detect bacterial and fungal pathogens in febrile neutropenia. J Clin Microbiol..

[14] Rivera MC, Lake JA (2004). The ring of life provides evidence for a genome fusion origin of eukaryotes. Nature..

[15] Liesenfeld O, Lehman L, Hunfeld KP, Kost G (2014). Molecular diagnosis of sepsis: New aspects and recent developments. Eur J Microbiol Immunol (Bp)..

[16] Trung NT (2016). Enrichment of bacterial DNA for the diagnosis of blood stream infections. BMC Infect Dis..

[17] Mervyn Singer CSD (2016). The Third International Consensus Definitions for Sepsis and Septic Shock (Sepsis-3). JAMA..

[18] Brun-Buisson C, Meshaka P, Pinton P, Vallet B, Group ES (2004). EPISEPSIS: a reappraisal of the epidemiology and outcome of severe sepsis in French intensive care units. Intensive Care Med..

[19] Varani S (2009). Diagnosis of bloodstream infections in immunocompromised patients by real-time PCR. J Infect..

[20] Suberviola B (2016). Microbiological Diagnosis of Sepsis: Polymerase Chain Reaction System Versus Blood Cultures. Am J Crit Care..

[21] Lehmann LE (2008). A multiplex real-time PCR assay for rapid detection and differentiation of 25 bacterial and fungal pathogens from whole blood samples. Med Microbiol Immunol..

[22] Dierkes C (2009). Clinical impact of a commercially available multiplex PCR system for rapid detection of pathogens in patients with presumed sepsis. BMC Infect Dis..

[23] Louie RF (2008). Multiplex polymerase chain reaction detection enhancement of bacteremia and fungemia. Crit Care Med..

[24] Westh H (2009). Multiplex real-time PCR and blood culture for identification of bloodstream pathogens in patients with suspected sepsis. Clin Microbiol Infect..

[25] Chang SS (2013). Multiplex PCR system for rapid detection of pathogens in patients with presumed sepsis - a systemic review and meta-analysis. PLoS One..

[26] Josefson P (2011). Evaluation of a commercial multiplex PCR test (SeptiFast) in the etiological diagnosis of community-onset bloodstream infections. Eur J Clin Microbiol Infect Dis..

[27] Dellinger RP (2013). Surviving sepsis campaign: international guidelines for management of severe sepsis and septic shock: 2012. Crit Care Med..

[28] Castellanos-Ortega A (2010). Impact of the Surviving Sepsis Campaign protocols on hospital length of stay and mortality in septic shock patients: results of a three-year follow-up quasi-experimental study. Crit Care Med..

[29] Tat Trung N (2018). Clinical utility of an optimised multiplex real-time PCR assay for the identification of pathogens causing sepsis in Vietnamese patients. Int J Infect Dis..

[30] Klaschik S, Lehmann LE, Raadts A, Hoeft A, Stuber F (2002). Comparison of different decontamination methods for reagents to detect low concentrations of bacterial 16S DNA by real-time-PCR. Mol Biotechnol..

