# Supplementary material for: PCR-based Sepsis@Quick test is superior in comparison with blood culture for identification of sepsis-causative pathogens
Source: Sci Rep. 2019 Sep 20;9:13663. doi: 10.1038/s41598-019-50150-y (PMC6754458; doi:10.1038/s41598-019-50150-y)
Supplement: Supplementary file 1 — Supplementary information [file 41598_2019_50150_MOESM1_ESM.docx]

**Supplementary Information’s for**

PCR-based Sepsis@Quick test is superior in comparison with blood culture for identification of sepsis-causative pathogens

Ngo Tat Trung^1,2,4,*,&^, Nguyen Sy Thau^1,3^, Mai Hong Bang^1,&^, Le Huu Song^1,3,*,&^

^1^Vietnamese-German Center for Medical Research (VG-CARE), 108 Military Central Hospital

^2^Centre for Genetic Consultation and Cancer Screening, 108 Military Central Hospital,

^3^Faculty of Tropical and Infectious Diseases, 108 Military Central Hospital

^4^Department of Molecular Biology, 108 Military Central Hospital

^&^These authors contributed equally to this work

**Corresponding authors**

**Assoc. Prof. Dr. Le Huu Song, MD, PhD**

108 Military Central Hospital, No 1, Tran Hung Dao Street,

Hai Ba Trung District, Hanoi, Vietnam, Tel. +84 69 698713,

**Email.** [lehuusong@108-icid.com](mailto:lehuusong@108-icid.com)

**Supplementary table 1: Primer and probes used in this study**

| **Bacterial group/ strain** | **Primer/probe 5'-3'** | **Accession Nr./ Target genes** | **Reference** |
| --- | --- | --- | --- |
| *E. coli* | GGCGAGAAACTGGCGATCCTTA | *Deoxyxylulose 5-phosphate* | [^1^](#_ENREF_1) |
|  | CGCTTCATCAAGCGGTTTCACA |  |  |
|  | FAM-TTTGGTACGCTGATGCCAGACG-BHQ1 |  |  |
| *K. pneumoniae* | CCGCGGACTATCTCGACTATAT | *AB106869* | Developed in house |
|  | CGATGGCATTATTGGGCGTAAATT |  |  |
|  | FAM-CGCTGGGCTTAATGACGATGGTATTTCCAGTGAT-BHQ1 |  |  |
| *N. meningitidis* | GCTGCGGTAGGTGGTTCAA | HQ437689/ctrA | [^2^](#_ENREF_2) |
|  | AATGGCTTCAGAAAGCGATAAGCCTCT |  |  |
|  | FAM-CTGACTCAGGCTTCCCGTAACGCTAAC-BHQ |  |  |
| *P. aeruginosa* | TACGGGAGGCAGCAGT | *16srRNA* | [^3^](#_ENREF_3)^,^[^4^](#_ENREF_4) |
|  | TATTACCGCGGCTGCT |  | [^3^](#_ENREF_3)^,^[^4^](#_ENREF_4) |
|  | Joe-GGAAGGGCAGTAAGTTAATACCTTG-BHQ |  |  |
| *A. baumannii* | TACGGGAGGCAGCAGT | *16srRNA* | [^3^](#_ENREF_3)^,^[^4^](#_ENREF_4) |
|  | TATTACCGCGGCTGCT |  |  |
|  | FAM-ATACCTAGAGATAGTGGACGTTACTC-BHQ |  |  |
| *Staphylococcus* spp*.* | CCGTGTTGAACGTGGTCAAATC | *TufA* | Developed in house |
|  | GCAACACCACGTAATAA(T/A/C)GCACC |  |  |
|  | Joe-TGTTGTCACCAGCTTCAGCGTAGTCTAATAATTTACG-BHQ1 |  |  |
| *S. aureus* | GAT TGA TGG TGA TAC GGT | *S. aureus Nuc gene* | [^5^](#_ENREF_5) |
|  | CAA GCC TTG ACG AAC TA |  |  |
|  | FAM-TGTACAAAGGTCAACCAATGACATTYAGA-BHQ |  |  |
| *Streptococcus* spp*.* | CAGC(A/T)CTTAAAGCTCTTGAAGG | *Tuf* | Developed in house |
|  | CGGAACATTTCAACACCAGTAAC |  |  |
|  | FAM-TGGWCGTGGTACWGTWGCTTCAGGACGTAT-BHQ |  |  |
| *S. pneumoniae* | ACGCAATCTAGCAGATGAAGCA | *AM113494/lytA* | [^6^](#_ENREF_6) |
|  | TCGTGCGTTTTAATTCCAGCT |  |  |
|  | FAM-TGCCGAAAACGCTTGATACAGGGAG-BHQ |  |  |
| *S. suis* | GTGTTCCATGGACAGATAAAGATGG | *GU223112/gdh* | Developed in house |
|  | CCGAGGAACTTCAAGATGGA |  |  |
|  | FAM-CCAAGTCAACCGTGGCTACCGTGTTCAGT-BHQ |  |  |
| *Enterococcus* spp*.* | TTTTTACTTTGTTCAGTTTTGAGAGGTT | *16srRNA* | Developed in house |
|  | AAGAAAATAAGCAATTGAACTTATTAAAAA |  |  |
|  | FAM- CAAACCGAGAACACCGCGTTGAAT -BHQ |  |  |
| *Fusobacterium sp,* |  |  |  |
| Tr-Fuso-F | CAACCAT TACT T TAACTCTACCATGTTCA | *16srRNA* | [^7^](#_ENREF_7) |
| Tr-Fuso-R | GTTGACTTTAC(A/T)GA(A/G)GGAGATTATGTAAAAATC | *16srRNA* | [^7^](#_ENREF_7) |
| Tr-Fuso-probe2 | FAM-TCAATTTCAGCAACTTGTCCTTCTTGATCTTTAAATGAACC-BHQ1 | *16srRNA* | [^7^](#_ENREF_7) |
| *Bacteriodes sp* |  |  |  |
| Tr-Leu-3 | 5′-CACTTGACTGTTGTAGATAAAGC-3′ | *leuB* gene of the *B. fragilis* | [8](#_ENREF_8) |
| Tr-Leu-4 | 5′-CATCTTCATTGCAGCATTATCC-3′ | *leuB* gene of the *B. fragilis* | [8](#_ENREF_8) |
| [Tr-Leu-Probe](http://jcm.asm.org/content/51/5/1593/T1.expansion.html#fn-2) | FAM-TGTGCTTGCTTCCAGTCGTCTATG-BHQ3′ | *leuB* gene of the *B. fragilis* | [8](#_ENREF_8) |

**Supplementary Figure 1**: SOFA score diffentiation upon Sepsis@quick diagnosis results.

The right panel: the difference of SOFA score in whole study cohort between subgroups with Sepsis@quick positive (n=83, Median score = 7, IQ: 4- 10) versus subgroup with Sepsis@quick negative (n= 61, Median score = 8, IQ: 4- 10). The right panel: Difference of SOFA score between in the sub-cohort of patients with blood culture negative (n= 95) between subgroups with (blood culture negative but Sepsis@quick positive, Median score = 6, IQ: 4- 9.75) versus (blood culture negative and Sepsis@quick negative group, Median score = 8, IQ: 4- 10).

**Reference**

1. Song, Y.*, et al.* Nuclease-assisted suppression of human DNA background in sepsis. *PLoS One* **9**, e103610 (2014).

2. Taha, M.K.*, et al.* Interlaboratory comparison of PCR-based identification and genogrouping of Neisseria meningitidis. *J Clin Microbiol* **43**, 144-149 (2005).

3. Horz, H.P., Vianna, M.E., Gomes, B.P. & Conrads, G. Evaluation of universal probes and primer sets for assessing total bacterial load in clinical samples: general implications and practical use in endodontic antimicrobial therapy. *J Clin Microbiol* **43**, 5332-5337 (2005).

4. Rohozinski, J. Compositions and methods for detecting and identifying bacteria Vol. WO2013096733 A1 (US, 2013).

5. Anna-Maria Costa, Ian Kay & Palladino, S. Rapid detection of mecA and nuc genes in staphylococci by real-time multiplex polymerase chain reaction. *Diagn Microbiol Infect Dis* **51**, 13-17 (2005).

6. Sakai, F.*, et al.* Single-plex quantitative assays for the detection and quantification of most pneumococcal serotypes. *PLoS One* **10**, e0121064 (2015).

7. Mima, K.*, et al.* Fusobacterium nucleatum and T Cells in Colorectal Carcinoma. *JAMA oncology* **1**, 653-661 (2015).

8. Papaparaskevas, J.*, et al.* Comparative evaluation of conventional and real-time PCR assays for detecting Bacteroides fragilis in clinical samples. *J Clin Microbiol* **51**, 1593-1595 (2013).
